# Clinical relevance of activities meaningful to parents of preterm infants with very low birth weight: A focus group study

**DOI:** 10.1371/journal.pone.0202189

**Published:** 2018-08-28

**Authors:** Mona Dür, Victoria Brückner, Christiane Oberleitner-Leeb, Renate Fuiko, Barbara Matter, Angelika Berger

**Affiliations:** 1 Department of Pediatrics and Adolescent Medicine, Division of Neonatology, Pediatric Intensive Care and Neuropediatrics, Medical University of Vienna, Austria; 2 Department of Health Sciences, Master Degree Programme of Applied Health Sciences and Bachelor Degree Programme of Occupational Therapy, IMC University of Applied Sciences Krems, Austria; 3 Department of Pediatrics and Adolescent Medicine, Division of Neonatology, Wilhelminenspital, Vienna, Austria; Universidade do Porto, PORTUGAL

## Abstract

**Introduction:**

Parents have a major impact on the outcome of health care of preterm infants. Parents’ engagement in meaningful activities could have an impact on their own health and wellbeing and therefore be relevant in neonatal intensive care. The aim of this study was to explore meaningful activities of parents of very low birth weight (VLBW) preterm infants with the purpose to further the understanding of their clinical relevance and to foster their consideration in clinical practice and research of neonatal intensive care.

**Methods:**

A total of 36 parents of preterm infants born prior to complete 37 weeks of gestation with VLBW (≤1.500 grams) were asked to participate in a focus group interview. Interview transcripts were used to analyse the content of the focus group interviews using meaning condensation method by Steinar Kvale.

**Results:**

Thirty-six parents participated in a total of twelve focus groups. Parents reported that the meaning of certain activities changed due to preterm birth. Meaningful activities, like bathing the baby and gardening, could foster a transition from a feeling of parental immaturity to a feeling of maturity, following health care instructions to possessing health care skills, and a functioning-only state to a balance of activities.

**Conclusions:**

In neonatal intensive care, nurses contribute to delivering parental education and thereby facilitate experiences of being a mature parent and of possessing health care skills. Occupational therapy could be used to help re-engage in meaningful activities and maintain a balance of activities in parents of VLBW preterm infants.

## Introduction

Eleven percent of live births worldwide (15 million per year) are preterm, defined as born prior to 37 completed weeks of gestation [1]. The main goals of neonatal intensive care are survival and prevention of prematurity-associated complications and long-term impairment. Especially, preterm infants with very low birth weight (< 1500 grams) are at increased risk for adverse neurodevelopmental outcomes [2, 3] and neonatal death [4]. Consequently, parents of preterm infants spend several months at the neonatal intensive care unit, constantly worrying about their infants’ survival, health and wellbeing, having to face the unexpected [5] and struggling with the impossibility to satisfy the urge to stay with the infant at all times [6].

Several studies highlight the importance of parents’ sensitive and compassionate caregiving for a healthy development of their children [7, 8]. Moreover, parents were found to considerably impact the health and outcome of health care interventions of their children [9]. In neonatal care the developmental care framework is used to involve parents in numerous activities to promote the health of their preterm infants [10]. For example, they change diapers, provide skin-to-skin contact and engage in feeding activities [11–13]. Furthermore, there is increasing evidence for the effectiveness of developmental and family-oriented pediatric health care [14, 15].

Parental activities in the neonatal intensive care unit (NICU) have been explored previously. For example, parents of preterm infants experienced challenges of coping with anticipated versus real activities and of transforming the meaning of parental activities [16]. However, activities other than parental activities could be meaningful to parents of preterm infants and clinically relevant and thereby an important outcome [17, 18]. Furthermore, there might be a link between activities meaningful to parents carried out outside the NICU and the infants’ health.

However, parental involvement in neonatal care could be related to experiences of stress [19] and overextension and to restricted engagement in personally meaningful activities. According to occupational science, meaningful activities include physical, mental, social and rest activities, as well as productivity, leisure and self-care activities. Meaningful activities are generally positive qualities of subjective experiences associated with human action or doing [20]. The meaning of these activities is determined by the individuals themselves and their social environment [21].

Previous studies provide initial evidence for a potential link between engagement in meaningful activities and parental and child health and wellbeing. For example, the engagement in meaningful activities was positively associated with health and wellbeing in mothers of preterm infants [22]. Furthermore, studies have shown that child health and wellbeing impacts parental engagement in meaningful activities [23, 24]. Additionally, associations between parental and child health and wellbeing have been proven [25, 26].

Considering these interrelations, parental engagement in personally meaningful activities could be linked to child health and wellbeing. It might be useful for professionals working in neonatal care to better understand the importance of meaningful activities of parents of preterm infants.

The aim of this study was to explore meaningful activities of parents of very low birth weight (VLBW) preterm infants with the purpose to further the understanding of their clinical relevance and to foster their consideration in clinical practice and research of neonatal intensive care.

## Methods

The researchers employed a qualitative study design to generate knowledge and an in-depth understanding of the meaning of certain activities for parents of VLBW preterm infant. Qualitative research designs can be used to explore the experience of a certain health condition from the perspectives of patients, caregivers and health professionals [27, 28]. The use of a phenomenological approach allows an exploration of the experiences and perspectives from a first-person point of view, who really have a lived experience of the phenomenon [29], such as being a parent of a preterm infant [30]. However, most of these studies used individual interviews, which might be restricted to the content which is directly asked by the interviewer and/or raised by the interviewee [31, 32]. Focus group interviews are lively discussions of people experiencing the same life event, situation or phenomenon and provide a possibility to gain an insight into the experiences and perspectives on a defined field of interest [31, 33].

### Participants

Parents who met the inclusion criteria were asked by the researchers for participation. Inclusion criteria for the study were: (1) being a parent of an infant born alive prior to complete 37 weeks of gestation and with VLBW (≤ 1.500 grams), (2) without a history of psychiatric and/ or neuro-motor disease and (3) with sufficient German reading and speaking skills. Furthermore, for inclusion, parents had to participate within six months after the discharge of their VLBW preterm infant(s). Exclusion criteria were (i) death of the preterm infant, (ii) insufficient German reading and speaking skills and iii) proven psychiatric and/ or neuro-motor disease.

Potential participants were recruited from parents of VLBW preterm infants treated at the Division of Neonatology, Pediatric Intensive Care and Neuropediatrics of the Medical University of Vienna, Austria in 2015. The researchers screened the medical records of the VLBW preterm infants to identify potential participants. The first author (MD), unknown to the parents, recruited eligible parents by phone or personally in the ward. Eligible parents were informed about the study purpose and data collection and were invited for participation.

Furthermore, for inclusion, parents had to participate within six months after the discharge of their VLBW preterm infant to ensure the currency of their experiences. We aimed to include a total of 36 parents following the suggestions for saturation sampling of focus group interviews [34]. Out of 172 candidate parents (86 mothers and 86 fathers) of VLBW preterm infants, twenty-six parents (15%) were not asked for participation for the following reasons: their own health condition (e.g. depression), the fact they did not answer the phone, or data collection was completed earlier with mothers than it was with fathers. The remaining 146 parents (85%) were asked to participate, among which 52 (30%) agreed and 94 (55%) refused to participate. Sixteen (9%) of the parents who originally agreed to participate could not keep the appointment for various reasons.

### Data collection

The focus group interviews were conducted in a private room in the intermediate care unit at the General Hospital/Medical University of Vienna. Participants who were couples participated separately. The number of participants per focus group varied from three to five. The small group sizes resulted in less time required and therefore took into account parents’ restricted time resources. Besides, small focus group sizes have been found to enable expressions of diversity and disagreement of perspectives and viewpoints [35]. Participants were asked to choose one of three different time points for the interviews. Most focus groups included mothers or fathers exclusively. Only two groups were mixed, due to restricted time resources of the parents.

The focus group interviews were led by a moderator (MD) skilled and experienced in conducting and analyzing focus group interviews. Participants were informed about the study purpose and procedures. Participants were asked to talk about their experiences related to their activities. They were encouraged to describe and discuss any experience and diverse opinions, even though they would consider them as unexceptional [31]. The focus group interviews followed an interview guide containing topics such as activities of daily living, meaning of activities before and after preterm birth and the maintenance of meaningful activities. All interviews were audio recorded and transcribed verbatim.

### Data analysis

Based on a phenomenological approach, the researchers conducted meaning condensation method [36] to analyze the content of the focus group interviews. The computer software Atlas.Ti was used [37]. In the beginning, the interview transcripts were read to get an overview of the whole data. Then the transcripts were coded according to meaning units which were expressions, several words or sentences on a common theme. The contents of the meaning units were summarized in sub-themes and grouped together (according to the content) in more comprehensive themes. Each theme encoded a certain meaning distinct to the other themes. Finally, the condensed meanings of the entire expressions were combined into descriptive statements [36]. To ensure rigor and trustworthiness of the analysis, participants were asked to read and give feedback to i) a German summary of the findings and ii) the manuscript of this article. Additionally, participants were separately informed about the pseudonym, original quote(s) and passage(s) referring to their statement(s). Supplemental written informed consent was obtained for the publication of these statements.

### Ethical considerations

The researchers obtained informed consent from all individual participants included in the study. The names, we are referring to in the original quotes at the results were changed to guarantee confidentiality of the participants. The ethical committee of the Medical University of Vienna specifically approved this study. The study is part of a larger project called “Occupational balance in informal caregivers—OBI-Care” [38].

## Results

A total of 36 parents of VLBW preterm infants participated in one of twelve focus groups between June and November 2015. Among these, two were mixed, five were with mothers, and another five were with fathers only. The mean duration of the focus group interviews was 102 minutes (± 15). All mothers were on maternity leave at the time of the interview, two of them were students, and another two were also self-employed. The participants were parents of a total of 35 VLBW preterm infants. Seventeen parents (47%) participated in the focus group interviews during the hospital stay and nineteen parents (53%) after discharge of their VLBW preterm infants. Demographic data of parents and VLBW preterm infants are depicted in Table 1.

**Table 1:** Demographic data of the parents and their infants.

	Female	Male	Total
**Parents’ characteristics**	18 (50%)	18 (50%)	36 (100%)
Mean age in years (±SD)	34 (±7)	38 (±6)	36 (±7)
Educational level^a^ Primary education	1 (6%)	3 (17%)	4 (11%)
Lower secondary education	4 (22%)	5 (28%)	9 (24%)
Upper secondary education	4 (22%)	2 (11%)	6 (16%)
Post-secondary non-tertiary education	1 (6%)	3 (17%)	4 (11%)
Short-cycle tertiary education	3 (17%)	2 (11%)	5 (14%)
Bachelor’s or equivalent level	5 (28%)	1 (5%)	6 (17%)
Master’s or equivalent level	0 —	2 (11%)	2 (6%)
Employment status^b^ Student	2 (11%)	1 (6%)	3 (8%)
Parental leave	18 (100%)	0 —	18 (50%)
Self-employed	2 (11%)	1 (6%)	3 (8%)
Employed	1 (6%)	15 (83%)	15 (41%)
Independent contractor	1 (6%)	1 (6%)	2 (6%)
unemployed	0 —	1 (6%)	1(3%)
Professional groups^c^			
Legislators, senior-officials and managers	3 (17%)	6 (33%)	9 (25%)
Professionals	6 (33%)	2 (11%)	8 (22%)
Technicians and associate professionals	0 —	5 (28%)	5 (14%)
Clerks	4 (22%)	0 —	4 (11%)
Service workers and shop and market sales workers	4 (22%)	5 (28%)	9 (25%)
Elementary occupations	1 (6%)	0 —	1 (3%)
Multiple birth	6 (33%)	5 (28%)	11 (31%)
First time parents	12 (67%)	13 (72%)	25 (69%)
**Children’s’ characteristics**	13 (37%)	22 (63%)	35 (100%)
Mean hospital stay in days (±SD, range)	214 (±71, 81–352)		
Mean APGAR 5 (±SD, range)	9 (±1, 4–9)		
Mean gestational age (±SD, range)	27+2 (±3, 23+4–33+5)		
Mean birthweight in gram (±SD, range)	1013 (±278, 550–1500)		
Preterm birth complications	BPD/CLD 5 (15%)	NEC 3(9%)	IVH IV 1(3%)
	ROP I 1 (3%)	ROP II 5 (14%)	ROP III 1(3%)

^a^ = Levels of the International Standard Classification of Education,

^b^ = Major groups of the International Classification of Occupations,

^c^ = Multiple answers allowed, BPD/CLD = Bronchopulmonary dysplasia or chronical lung disease, IVH = intraventricular hemorrhage, NEC = necrotizing enterocolitis, ROP = retinopathy of prematurity, SD = Standard deviation.

### Experiences of parents of VLBW preterm infants

A total of seven activity-related themes were found to be important to parents of VLBW preterm infants. These were: a change in the meaning of their activities, a transition from a feeling of parental immaturity to a feeling of maturity, from following health care instructions to possessing health care skills, and a transition from a functioning-only state to a balance of activities. In the following paragraphs, the themes are described as statements and substantiated by original quotes:

Parents reported a change in the meaning of their activities. For example, Cornelia, a 34-year-old mother of a boy born at a gestational age of 30+4 weeks said: „*Small things*, *daily routines were very important to me*. *Activities such as changing the nappy*, *bathing* [the baby]. *What I didn’t expect is that nothing else really mattered to me*. *Even though I had to run a household and we own a company*”(Cornelia, 34 years old, son [30+4, discharged], 5:182–5). A 43-year-old father of a girl with a gestational age of 25+2 weeks, Thomas, described a change in the meaning of gardening in the following way: “*Gardening*. *It’s grounding me*. (…) *It’s growing and green*. *It blossoms*, *grows and thrives*, *this is just enjoyable*. *It’s metaphoric also*. *The little one* [baby] *is fed also*, *and grows and thrives*. *This is certainly so lovely*” (Thomas, 43 years old, daughter [25+2, discharged], 3:396–411). In addition, cooking and eating together with their partner began to mean nurturing the loved ones, which was also a symbol for their loving feelings while struggling with the various attempts and activities to nourish their baby.

Furthermore, parents reported that meaningful activities had an impact on their transition of becoming a parent of a preterm infant. Meaningful activities could foster their transition from a feeling of parental immaturity to a feeling of maturity, from following health care instructions to possessing health care skills, and their transition from a functioning-only state to a balance of activities.

For example, parents frequently reported that they were not parents at the beginning, and that meaningful activities could help them to develop feelings of parenting and attachment. Table 2 gives an example for the description of the transition from a feeling of parental immaturity to a feeling of maturity. The original illustrative quotes include anonymized information on the parents and their VLBW preterm infants, focus group number and transcript lines.

**Table 2:** Statement I.

Activities foster a transition from a feeling of parental immaturity to a feeling of maturity.
**Being an immature parent:** “*I don’t feel complete*. *I am not a mother yet*, *not fully a mother*. *It is just too early*” (Lea, 34 years old, daughter [25+0, in-patient], 2:663–5). “*At the beginning I felt like a visitor*. *It took ridiculously long until I realized that they were my children*. *I came* [to the hospital], *did a few things and went home*. *It was as if I were just a visitor*, *as if they weren’t my children*” (Tim, 40 years old, twin daughter and son [27+2, discharged], 3:722–6).
**Becoming a mature parent:** “*To tell the truth*, *we had two and a half months training to get to know the kids*” (Tim, 40 years old, twin daughter and son [27+2, discharged], 3:711). “*It was important for me to be there*, *to take part* [in caring for the child], *so that I realized that I was the mother of this tiny child*” (Ruth, 40 years old, son [28+0, discharged], 2:367–9).
**Being a mature parent:** “*They* [health professionals] have *started asking me*: *what do you think*, *how should we position him*, *and which positions does he like*? *You don’t feel so helpless or just having to follow to their instructions*. (…) *I have started experiencing myself as his mother*” (Simone, 37 years old, son [25+2, discharged], 7:357–62). “First, y*ou are involved in* [neonatal] *care and you are somehow supervised*. *Then you get home and you already know how it* [baby care] *works*” (Max, 39 years old, son [30+4, discharged], 3:721–7).

**Bold** = sub-themes, *italic* = original illustrative quote, (pseudonym, age, daughter or son, [gestational age at birth, in-patient or discharged at the time of the focus group interview], number of focus group: transcript line(s)).

Furthermore, parents were found to gain health care skills by engaging in meaningful activities such as talking to health care professionals, bathing and feeding the child and by requiring feedback from health professionals, especially from nurses. The possessing health care skills allowed them to act independently and self-assured, often after a long period of guidance from the health professionals. Table 3 contains selected original quotes as examples of their transition from following health care instructions to possessing health care skills.

**Table 3:** Statement II.

Activities foster a transition from following health care instructions to possessing health care skills.
**Following health care instructions:** “*I thought I would only do it wrong*. *But*, *they* [health professionals] *however*, *encouraged me*. *And my daughter*, *I have a 19*-year-old *daughter*, *said*: *‘Bodily contact that’s what’s really important*. *So*, *I thought if this is really important* [I’ll follow the instructions]” (Theo, 37 years old, son [29+3, discharged], 8:53–8). “*Since we’ve been told that this is so important we cuddle* [the baby] *as much as possible*,” (Gerald, 32 years old, son [24+0, in-patient], 9:154–6). **“***From the very first second*, *they* [health professionals] *are your guides and over many weeks*, *you feel totally dependent*” (Cornelia, 34 years old, son [30+4, discharged], 5:1246–8).
**Gaining health care skills:** *“We asked a lot*, *we wanted to understand* [the decisions of the health professionals]. (…) *Talking with them*, *that’s the way you grow into it”* (Max, 39 years old, son [30+4, discharged], 3:454–5). “*During bradycardia*, *you are supposed to massage the child*” (Luzia, 33 years old, twin sons [28+4, in-patient], 1:1230–3). *“I read one and the same story to him night and day*. (…) *Finally*, *I started to change the diapers inside the incubator”* (Simone, 37 years old, son [25+2, discharged], 7:13–8).
**Having health care skills:** “*At night*, *when it* [the monitor] *beeps—it beeps when he* [the baby] *stops breathing–we* [immediately] *check what’s going on*. *Sometimes there are incidental interferences*. *Or it beeps because the electrode is on the romper*” (Lukas, 29 years old, son [26+6, discharged], 4:42–4). “*They* [the health professionals] *ask*: *Have they* [twins] *completely drunk up*? *In the back of my mind I was thinking*: *If not*, *they* [twins] *would get it* [the milk] *via the feeding tube”* (Marie, 37 years old, triplet daughters [33+5, discharged], 1:303–5). *“And then they wouldn’t be able to go home*. (…) *They have to be off the feeding tube for a few days before they are allowed to go home*” (Luzia, 33 years old, twin sons [28+4, in-patient], 1: 306–8).

**Bold** = sub-themes, *italic* = original illustrative quote, (pseudonym, age, daughter or son, [gestational age at birth, in-patient or discharged at the time of the focus group interview], number of focus group: transcript line(s)).

Additionally, parents described that they were in a status where they were just functioning. A status in which they just did, what had to be done and in which own needs weren’t important at all. In the functioning-only status it was most important to do everything in ones’ power to avoid further deteriorations of the current situation. Previous meaningful activities were abandoned for a while, until they started to regain a balance of activities. For example, a balance of activities which had to be done and activities which they wanted to do. Table 4 presents quotes which demonstrate the transition from a functioning-only state to a balance of activities.

**Table 4:** Statement III.

Activities foster a transition from a functioning-only state to a balance of activities.
**Being in a functioning-only state**: “*For him* [the father], *it is an absolute exceptional situation*. *I think he is just functioning from day to day*. *Yes*, *it cannot be described differently*. *At the moment*, *we are just functioning*, *getting along from one day to the next*, *somehow*” (Lea, 34 years old, daughter [25+2, in-patient], 2:438–9). “*When I get asked*: *How are you*? *I think*, [how am] *I*? *He* [the child] *is the one who needs to be okay*; *all* *I* *need is functioning at home*, *cope with things*, *while he needs to get better*. (…) *You know*, *you tend to forget about yourself*” (Saida, 50 years old, son [29+4, in-patient], 10:688–90).
**Regaining a balance of activities:** “*Playing PlayStation*, *for me it is a kind of mental break*. *I can really get away from it all and just be silly for one or two hours*. *If you are steadily running on a certain level* [of activity], *you need to unwind*” (Felix, 43 years old, daughter [28+4, in-patient], 8:218–22). “A *day at the hospital I find very strenuous*. *Even though*, *we just huddle together and feed and bath* [the baby]. *It is really exhausting*” (Luzia, 33 years old, twin boys [28+4, in-patient], 1:228–31). “*I allow myself a coffee*, *to take fresh airing and to soak up the sun*, *drink a cup of coffee in front of the hospital*. *That’s my time to relax*” (Tina, 31 years old, daughter [30+2, in-patient], 1:716–20).
**Being in a balance of activities:** “*I am an actor at an amateur theatre*. *This helps me to distract my thoughts and to think of something else*. [It helped me] *not to speak of the preterm birth*. (…) *Acting helped me to relax and to recharge my energy*. (…) *Rehearsals in the weekend were quite stressful*. *However*, *from a present-day perspective*, *it was somehow positive for me*” (Stefan, 44 years old, son [28+0, discharged], 4:275–83). “*Now we can move freely*. *We can change loci; we aren’t locked in the patient’s room anymore*. *We go out*, *visit relatives* (…) *or we attend mother-child-groups*” (Ruth, 40 years old, son [28+0, discharged], 2:398–401).

**Bold** = sub-themes, *italic* = original illustrative quote, (pseudonym, age, daughter or son, [gestational age at birth, in-patient or discharged at the time of the focus group interview], number of focus group: transcript line(s)).

## Discussion

The results of the study indicate that the meaning of activities for parents of VLBW preterm infants changes over time and that, meaningful activities can foster transition. More specifically, activities meaningful to parents of VLBW preterm infants were found to foster a transition from a feeling of parental immaturity to a feeling of maturity, from simply following health care instructions to possessing health care skills and from a functioning-only state to a balance of activities. Previous studies found that parents of preterm infants undergo a transition process, such as adjusting to preterm birth and to deviations from the parental role formerly imagined [5].

In the current study, parents of VLBW preterm infants reported about a change in the meaning of certain activities, such as gardening and cooking. For example, fathers reported that gardening became a meaningful activity during the hospital stay of their preterm infant. In addition, while cooking and eating was related to still one’s hunger, it became symbolic for nurturing the loved ones. An adaptation of the meaning of certain activities was reported previously in persons who experienced a loss of meaningful activities, due to their health condition, disability or a transition phase, such as the transition from employment to retirement [39, 40]. However, a change in the meaning of activities in parents of VLBW preterm infants was not reported before.

In the current study, meaningful activities were found to foster a transition from a feeling of parental immaturity to a feeling of maturity. This effect has been reported from parents of preterm infants previously. In a recent study on parental activities in the NICU, engaging in meaningful activities helped parents to overcome their feelings of unpreparedness [16] and to explicate a process of adaptation and the development of parental identity [16, 30]. Additionally, parents reported that the engagement in meaningful activities, like bathing the child, elicited feelings of parental confidence and attachment [41].

The findings indicate that engaging in meaningful activities, especially in child and health care activities, such as changing diapers and feeding the child, fostered a transition from simply following health care instructions to possessing health care skills. The acquisition of health care skills through the engagement in meaningful activities of parents of preterm infants was reported recently. For example, it was found that these activities encourage parents to increasingly check, interpret and respond to their preterm infant’s temperature and oxygen saturation, and to actively engage in the health care of their preterm infant [16]. Additionally, parents’ engagement in the care of their preterm infants was found to promote a feeling of autonomy [6].

Engaging in meaningful activities, such as having a walk or acting at an amateur theatre, was found to foster a transition from a functioning-only status to a balance of activities. Functioning, like family functioning, behavioral, cognitive, executive and motor functioning were explored in preterm infants and their parents [42, 43]. A balance of activities was explored in parents [44]—but not in the parents of preterm infants. In addition, to our knowledge, this is the first study reporting a transition from a functioning-only status to a balance of activity in parents of preterm infants.

The findings of the current study highlight the importance of various activities from the perspectives of parents of VLBW preterm infants. To our knowledge, meaningful activities other than childcare activities have not been considered in neonatal clinical practice. However, as described in the introduction, initial evidence has been gathered showing a potential link between engagement in meaningful activities and parental and child health and wellbeing [23, 24, 45]. Moreover, the therapeutic use of meaningful activities has been found to improve patient and caregiver health and wellbeing [46, 47]. Considering the potentially favorable effect of parents’ various meaningful activities on their preterm infants’ health and wellbeing, such activities could be clinically relevant in the context of neonatal care. Since evidence for this favorable effect is scarce, further research is needed.

There are no studies reporting health care interventions targeting a transition from a functioning-only status to a balance of activities in parents of preterm infants. Occupational therapists are part of the NICU team [48] and are using meaningful activities as a means and as an outcome of their interventions [49]. Therefore, we suggest occupational therapy [50] to support parents’ engagement in various meaningful activities, beyond those related to their child(ren) and transition towards a balance of meaningful activities. Furthermore, commonly used parent related outcomes of neonatal care, such as parental stress [51], could be complemented with other outcomes meaningful to parents of preterm infants, such as a balance of meaningful activities.

### Strengths and limitations

Qualitative research uses small sample sizes to generate meaning and to allow in-depth analyses of each case. The number of participants per focus group varied from three to five. The small group sizes resulted in less time needed and therefore considered parents restricted time resources. Small focus group sizes were found to enable expressions of diversity and disagreement of perspectives and viewpoints [35]. An exploration of the perspectives of parents of VLBW preterm infants of different hospitals and regions, as well as other data collection and analysis methods could have led to different findings. Various strategies were used to assure rigor and trustworthiness. Unlike other studies on parents of preterm infants [22], the current study explored the perspectives of both mothers and fathers.

## Conclusions

Activities beyond childcare activities were found to be meaningful to parents of VLBW preterm infants and to foster a transition to a feeling of parental maturity and a balance of activities. In neonatal intensive care, nurses contribute to delivering parental education and thereby facilitate experiences of being a mature parent and of possessing health care skills. Considering the link already found between meaningful activities and health and wellbeing, parents’ increased engagement in various meaningful activities could become an important objective for neonatal care and thus could become clinically relevant. Occupational therapy could be used to help re-engage in meaningful activities and maintain a balance of activities in parents of VLBW preterm infants.
